# A commentary on podiatry during the Covid-19 pandemic: Podiatry during the Covid-19 pandemic

**DOI:** 10.1186/s13047-020-00425-9

**Published:** 2020-10-15

**Authors:** Paul Chadwick, Lawrence Ambrose, Ross Barrow, Martin Fox

**Affiliations:** 1 The College of Podiatry, London, UK; 2 Manchester Local Care Organisation, Manchester, UK

## Abstract

**Background:**

The arrival of the novel coronavirus (SARS-CoV-2) has impacted the many aspects of modern life, especially, in the immediate term, the delivery of healthcare.

**Context:**

This commentary examines the profession of podiatry and how it has adapted and responded to the emerging crisis. It focusses on but is not exclusive to the position in the United Kingdom (UK) and the edicts and direction from the UK Government.

**Podiatry roles during the pandemic:**

It describes the role of podiatry in the pandemic and highlights the deployment of podiatry resources to fight the pandemic beyond traditional podiatric practice. It also looks at the shift from conventional consultation to digital solutions for managing patients in an effort to achieve the goals of maintenance of foot health whilst reducing the spread of the virus. The commentary summarises the emerging data related to a possible foot related presentation of the coronavirus.

**Conclusion:**

The podiatry profession proved its flexibility and adaptability during the pandemic, to adjust rapidly to ensure that patients were able to access treatment to reduce risk of infection, ulceration and amputation. Dermatological presentations on the feet have been associated with Covid-19 in adolescents as is often the case in viral infections. CPD webinars to support clinicians and manage and prevent the spread of Covid-19 have been widely disseminated along with algorithms to ensure that patients that need treatment are being treated appropriately. Podiatrists have embraced remote technology to ensure that patients are correctly and safely triaged and, signposted and given appropriate self-care advice. MSK podiatrists have the ability to play an intrinsic role within the post discharge rehabilitation pathway.

## Background

The novel coronavirus (SARS-CoV-2) continues to impact around the world and is not only an evolving current crisis but has the potential to impact healthcare provision moving forward in the short and medium term. Ultimately, it may change how healthcare services are delivered permanently as people build on positive changes made to delivering care in this challenging time but also due to the resources available in a post Covid-19 pandemic world.

Based upon our current understanding and knowledge we aim to examine how the Covid-19 pandemic has impacted foot health in the UK, how services have adapted and evolved and look at any evidence that it has a direct pathology within the feet.

## Context

Covid-19 has been classified by the World Health Organisation as a pandemic [1]. In many countries across the world, Covid-19 is placing unparalleled pressures on healthcare systems and disrupting patient care as all financial and human resources are mobilised to fight the virus [1].

In the UK, provision of many foot health services have been changed or temporarily reduced [2–4], as UK Governments attempt to limit non-essential contact and podiatrists are deployed to other parts of the healthcare system as part of the fight against the virus [5]. The professional body for podiatrists, the College of Podiatry in the UK has advised, that all routine or non-urgent treatments and assessments are delayed until further notice; however, this may be creating issues for the future. The role of the podiatrist in both pandemic and normal times has been recently highlighted [6]. Rogers et al. (2020) reported that podiatric care is associated with fewer diabetes-related amputations, Emergency Department visits, hospitalisations, length-of-stay, and reduced costs.

Podiatrists have a critical role to play during the pandemic [2–4], particularly in preventing diabetic and vascular foot ulceration, and related hospital admissions. Such admissions would add pressure to intensive care unit capacity. The Vascular Society of Great Britain and Northern Ireland have produced guidance which indicates that surgical reconstruction of a limb with peripheral arterial disease is less likely during the current crisis due to resources such as critical care capacity being used to manage Covid patients [7]. Podiatric care is also critical for those who are at increased risk from Covid-19, including adults over 70 years old and those with underlying health conditions such as diabetes, hypertension and obesity.

## Defining essential podiatry treatment

NHS England and NHS Improvement, the Welsh and Scottish Governments promptly produced guidance around which community services should be withdrawn during the pandemic [2–4]. Specific to the podiatry profession, the guidance states *“that high risk vascular/ diabetic foot clinics should proceed as normal, whilst elective, non-high- risk surgical procedures should be cancelled.”* Wherever possible digital technology and/or adopting remote triage as the default and delivering care and treatment remotely, based on clinical judgement is stipulated to provide advice and support to patients before home care is considered [2–4]. People with diabetes have a higher overall risk of infection that results from multiple perturbations of innate immunity. In a recent report from NHS England indicated that diabetes patients were of greater risk of mortality as a result of Covid-19 [8]. Consequently, there has been an upsurge of information relating to diabetes and Covid-19. Notably, the College of Podiatry in the UK produced an algorithm as an aid for independent practitioners which should be used in conjunction with NICE guidance NG19 [9].

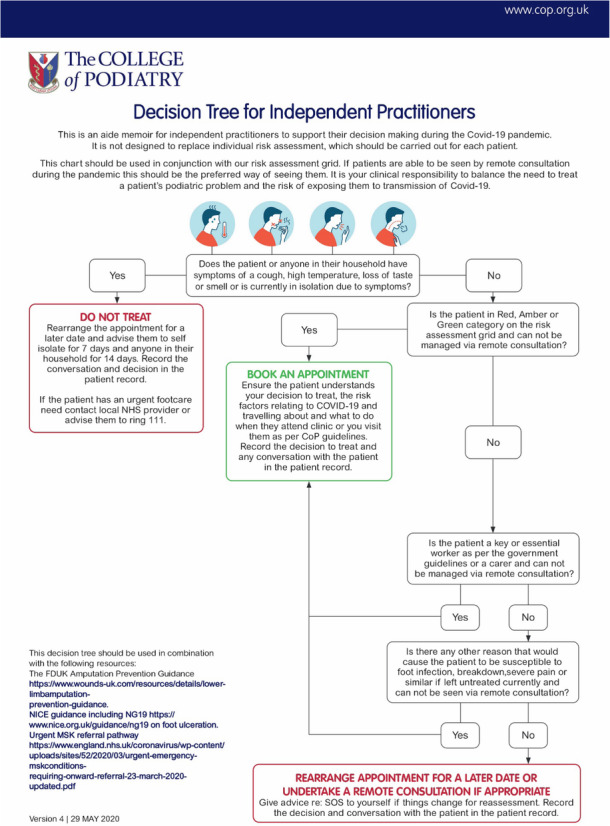



Key to lowering the mortality rates is through health systems across the world ensuring they have the maximum amount of Intensive Care Unit (ICU) beds with ventilator facilities. Acute wards have been restructured and staff redeployed to ensure this. It is therefore essential that any Podiatric procedures that can prevent admission to an acute ward should continue so that ICU capacity is maintained. For example, procedures such as managing serious foot infections, gangrene and trauma would reduce the requirement for patients to be admitted to secondary care [10]. In that context, the podiatrist has to rigorously assess the risk factor of the patient, as well as their presenting symptoms.

## Strategic redeployment of the podiatric workforce

While there has been recognition of the need to maintain limb preservation services such as multidisciplinary diabetic footcare teams, wound care and vascular access, the role of preventative care has been greatly affected. Many front line foot services both in state funded and private or independent practice have been reduced and/or suspended and much of the podiatric workforce has been redeployed to take up other health care roles in areas such as intensive care, tissue viability and rehabilitation.

The UK Government exempted independent Podiatry clinics as needing to close in the Covid-19 guidance on the closure of certain businesses and venues as part of further social distancing measures [11]. Podiatrists working in independent practice have risk assessed the appropriateness of remaining open for patients who may require urgent podiatric care but not preventative care. As the peak of the pandemic curve passes and recovery begins, the absence of preventative podiatric care over a prolonged period of time is likely to have a consequential effect on increasing foot ulceration and infection rates. Sepsis and amputation in those with a foot ulcer will also inevitably increase. If amputation rates increase there will be an increase in the requirement for post-amputation rehabilitation and prosthetics, yet with many services being redirected to manage the Covid-19 pandemic, there would be limited capacity for this. This coupled with a growing societal reluctance to attend hospital and clinic appointments which has been described in Italy [12] may potentially lead to a tsunami of foot related complications in the coming months and years.

The Vascular Society of Great Britain and Ireland have suggested that performing primary amputation may be more appropriate than performing complex vascular reconstructions to reduce prolonged hospital stays [7]. Many GPs and others view people with intermittent claudication as needing to be referred to vascular teams, anecdotally we have heard from vascular teams that their services remain open, but during the Covid-19 crisis not to refer people to acute vascular services with asymptomatic peripheral arterial disease or intermittent claudication (mild to moderate severity disease only) [7]. Accordingly, the role of ‘vascular capable’ podiatrists in triaging all suspected lower limb arterial disease from mild asymptomatic disease, through to severe / critical ischaemia is essential in supporting both primary care and secondary care vascular teams. This may be accomplished via telemedicine consultations where possible, and face to face assessments where severe or deteriorating symptomatic disease is likely, via history and limb assessment, including ABPIs, toe pressures, and foot to femoral pulse checks.

## Possible presentation of Covid-19 in the feet

Recent reports [13–17] have highlighted some of the suspected skin-related symptoms associated with Covid-19 infections, which demonstrated a chickenpox-like rash occurring on the toes of adolescents who have tested positive for the infection. As a new infection, healthcare professionals are still learning more about the effects of the disease but emerging reports are noticing that the skin can be affected with various rashes across the body and limbs. These include chickenpox like spots, red itchy areas (similar to chilblains) and urticaria (wheals). Many viral infections are known to produce skin changes, but we do not have any firm evidence that those being observed at the moment are associated with Covid-19 infections. As more data is collected from around the world, this may provide a clearer picture as it develops. This emergence of new evidence around Covid-19 presentations in the feet in rapidly changing circumstances, such as during a pandemic was described in the journal recently [18].

## Use of digital technology

The UK and wider international research community and industry have risen to the challenge for expertise, resource and technology to support clinicians, as well as help manage and prevent the spread of Covid-19. The International Working Group on the Diabetic Foot (IWGDF) supported by associations from around the world have developed a global repository for guidance and a forum for discussion on managing diabetic foot disease during this period [19].

D-Foot international have produced a number of educational webinars [20] to help support clinicians who maybe being asked to manage more complex presentations than is their usual routine.

In the UK, ‘Foot in Diabetes UK’, a group of experts in the management of diabetic foot disease, have published guidance to assist the identification of and management of people with critical/ limb threatening ischaemia or infection [10]. The aim of the guidance is to support all lower limb clinicians working within the UK health system during the Covid-19 situation in line with current best practice. The focus is on clinical assessment and decisions on urgent triage, referrals and access to High Risk Podiatry, Acute Vascular, Diabetic Foot, Infectious Diseases or Orthopaedic Teams, for potential life and limb salvage interventions.

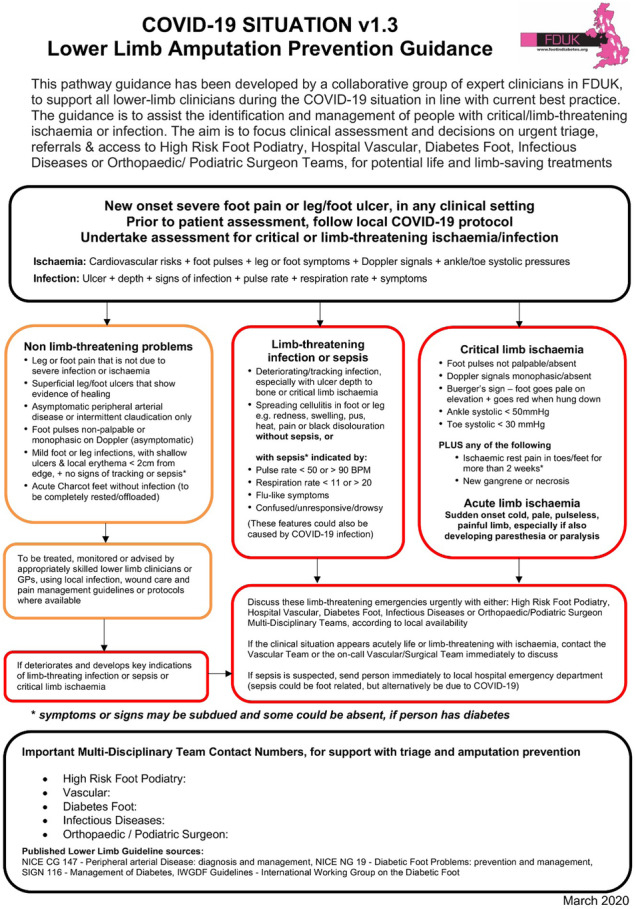



The College of Podiatry have also been providing clinical and professional support via webinars and web-based resources, and has produced guidance on remote consultations for podiatrists working across all sectors. Podiatrists across the world are embracing Technology Enabled Care including platforms such as Attend Anywhere and Near Me where appropriate and feasible. Podiatrists are taking all steps necessary to ensure any remote consultations are secure, encrypted and password protected; and all regular standards of professionalism are upheld when using technology (such as not answering calls/the door during consultations). At this critical time for the healthcare system, where resources are at a premium, an appropriate method of triaging patients to evaluate which assessments and treatments are essential, (as opposed to routine), is more important than ever. Rogers et al. (2020) writing in Journal of the American Podiatric Association, have published a triage system which will “drive the site and urgency of podiatric care,” during the pandemic [6].

## Rehabilitation

Podiatry has a vital role to play in the rehabilitation of Covid-19 patients, working in and leading multidisciplinary teams to support people to regain mobility and improved foot and lower limb health.

The four nations’ statement, *Allied Health Professionals’ role in rehabilitation during and after COVID-19* (May 2020) [21] identified four main population groups who will have an increased need for rehabilitation both during and after the pandemic: -.

1. Those recovering from Covid-19
2. Those whose health and function are at risk due to pauses in planned care
3. Those who have avoided accessing health services during the pandemic and are therefore at increased risk of ill-health due to delays in diagnosis and subsequent treatment
4. Those for whom the lockdown has caused physical and mental challenges

Podiatrists are able to reduce pressure on, and provide care to the following four groups:

*Those recovering from Covid-19* - undertaking neurological and functional assessment to identify lower limb muscle weakness and balance/mobility impairment, provide bespoke care packages to reduce the risk of foot ulceration and amputation and provide pressure ulcer prevention education for carers and patients.

*Those whose health and function are at risk due to pauses in planned care-* The majority of elective foot and ankle surgeries have been postponed due to the pandemic. Podiatric surgeons will be supporting people with the interventions required to ensure they are able to regain full function and mobility.

*Those who have avoided accessing health services during the pandemic and are therefore at increased risk of ill-health due to delays in diagnosis and subsequent treatment-* Those who have been unable to access care during the lockdown, who may potentially be suffering with complications, will require podiatry services as a matter of urgency.

*Those for whom the lockdown has caused physical and mental challenges*- Improving public health is a critical part of a podiatrist's role, as the profession takes a holistic approach to keeping the population healthy, mobile and active.

Whilst the majority of musculoskeletal (MSK) podiatry clinics have been reorganised into virtual telephone/ video clinics during the pandemic, the role of MSK specialist podiatrists is crucial in the wider rehabilitation team to ensure people can be discharged safely to their own homes. Podiatrists can support discharge teams and assess people’s mobility and activities of daily living; this role can also include supporting ward discharge teams. In community, after discharge, these roles are vital to support occupational therapy and physiotherapy colleagues to assess patients’ ability to live independently and ensure falls and fragility assessments are carried out.

## Conclusion

The Covid-19 pandemic has changed the face of healthcare, whether this is a positive or negative we shall wait and see in time. Podiatrists have made a step change too with many taking a lead on triaging all foot and lower limb conditions (whether wound, vascular, musculoskeletal or dermatological) as well as being redeployed due to their transferable skills to support ICUs and frontline health system staff. Complacency, however, is not to be accepted. The challenge of the current crisis is hard to deal with, especially when we cannot predict the recovery and consequent morbid and chronic foot problems that may surface over the next 12 months. The recovery plan for foot health services needs to be in place for management of all those foot wounds that have not had the attention from podiatrists that they would normally receive. Even more so for all those people who will have missed eight to twelve weeks of usual care and will be in need of simpler aspects of care such as corn and callus debridement to prevent ulceration and chronic wounds. Podiatrists will therefore continue to be an essential part of this. Moving forward, we as a profession - should seek to monitor and record the impact of Covid 19 upon foot health service provision and foot health pathology. This should form part of an overall approach to a recovery plan and in alignment with the results of the emerging evidence base. It should also inform part of our “preparedness” for a second wave and future pandemic planning.

## Data Availability

Not applicable.
